# Deficits in Emotion Regulation Partly Mediate the Relation Between Sleep Problems and Depressive Symptoms in Adolescent Inpatients With Depression

**DOI:** 10.3389/fpsyt.2021.622833

**Published:** 2021-05-17

**Authors:** Inken Kirschbaum-Lesch, Martin Holtmann, Tanja Legenbauer

**Affiliations:** LWL-University Hospital for Child and Adolescent Psychiatry and Psychotherapy Hamm, Ruhr-University Bochum, Bochum, Germany

**Keywords:** sleep problems, depressive symptoms, deficits in emotion regulation, adaptive and maladaptive strategies, adolescents

## Abstract

Sleep problems are a risk factor for the development of depressive disorders and influence the severity and treatment of depressive symptoms negatively. To enhance treatment for depression in young people, it is important to advance the understanding of the relationship between sleep problems and depressive symptoms. Since deficits in emotion regulation are discussed as possible underlying mechanisms, the present study investigated the mediating effect of maladaptive and adaptive strategies for emotion regulation on the association between sleep problems and depressive symptoms. Emotion regulation strategies, depression and sleep quality were assessed via self-report in a large clinical sample of 602 adolescents (age 13–18 years) who reported clinically relevant symptoms of depression. The questionnaires were assessed at admission for inpatient psychiatric treatment. Correlation and mediation analyses were performed. There was a significant partial mediation effect (β = 0.554, *p* < 0.001, *R*^2^ = 0.527), indicating that sleep problems influenced depressive symptoms via the decreased use of adaptive strategies and the increased use of maladaptive strategies. Additionally, a direct effect of sleep problems on depressive symptoms emerged (β = 0.251, *p* < 0.001, *R*^2^ = 0.364). This cross-sectional study provides first indications that additional treatment modules focusing on sleep and ER skills in prevention and treatment programs for adolescents would be important steps. Longitudinal studies are needed to substantiate these results.

## Introduction

Depression in youth is a prevalent disease associated with a high risk of chronicity and functional impairment (1). Sleep problems are common and may aggravate the course of the disease (2, 3). Recent evidence outlines the importance of emotion regulation (ER) as a possible underlying mechanism of the association between sleep problems and depression (4, 5). Mood disorders were indirectly related to sleep problems through lower problem solving and greater rumination in a nationally representative adolescent US sample (5). Also longitudinal evidence emphasized the role of maladaptive ER as mediator between poor sleep quality and depression in adults with current and remitted depression as well as in healthy controls (4).

However, in adolescents with clinically relevant levels of depression empirical evidence is missing. Furthermore, the described studies miss to include adaptive strategies in contrast to maladaptive strategies when looking at the indirect association of depression with sleep problems through dysfunctional ER (6). The present study aims to fill these gaps. It seems to be relevant to examine the associations between sleep problems, dysfunctions in ER and depressive symptoms in clinical adolescents with depression to strengthen prior findings. The results may help to provide insights to inform treatment and prevention efforts in adolescent depression.

### Sleep and Depression

Around 75% of children and adolescents with depression report sleep problems, mainly in the form of insufficient sleep, non-restorative sleep and increased daytime sleepiness (7, 8). The natural circadian phase shift toward eveningness during adolescence (9) contrasts with social duties encouraging early waking (e.g., school/working timing). This social jetlag may lead to sleep loss and insomnia in many adolescents with consecutive mental health problems such as clinical depression (10). Sleep problems are not just a diagnostic criterion for depression according to the International Classification of Diseases (ICD-10), but are also a relevant risk factor for the development and maintenance of depressive disorders. In adolescents, a recent meta-analysis showed that shorter sleep duration increased the risk of mood deficits (OR = 1.55) (11). Another meta-analytic study revealed that people with sleep problems have a twofold higher risk of developing depressive disorders compared to people without sleep problems (2). In addition, sleep problems in adolescence are associated with more severe symptoms of depression, longer depressive episodes and suicidal thinking, and have a negative influence on the course of treatment (3, 12–14).

Two studies examined differences in sleep problems between healthy girls and boys (between 9 and 16 years) at low and high familial risk for depressive disorders (one parent with recurrent depression) (15, 16). They found significant differences between subjective reports of sleep disturbances (e.g., daytime sleepiness, difficulties falling/staying asleep) between both groups, especially in girls (16). The authors hypothesized that the high-risk adolescents had an increased risk to develop psychopathology such as depression due to cognitive and affective impairments which were caused by disturbed sleep (16). There are several studies that support these assumptions by (1) showing that sleep problems impair executive functioning skills such as acquisition, consolidation and recall of information (17) and (2) indicating that sleep problems increase negative affective states and decrease positive ones as well as impair emotion regulation processes (18).

Although the association between sleep problems and depressive symptoms is complex and seems to be bidirectional (19), a systematic review emphasizes that the impact of sleep problems on depression is much stronger and persistent than the other way around (20).

### Deficits in ER and Depression

ER refers to the ability to influence emotions in terms of their intensity, duration, timing and expression (21). In general, ER strategies are classified as adaptive and maladaptive according to their functionality (22). Adaptive strategies (e.g., cognitive reappraisal, acceptance) aim to reduce unpleasant emotional experiences and to maintain a person's well-being with regard to his/her goals and needs in the long term. Maladaptive strategies (e.g., rumination, suppression) are also able to reduce unpleasant emotions, but only in the short term. Thus, maladaptive strategies are often unable to maintain this inhibiting effect on unpleasant emotional experiences, and in the long term, such strategies counteract a person's well-being (23). The usefulness of ER strategies is context-dependent and cannot be generalized for every situation (24). Nonetheless, evidence suggests that the frequent use of maladaptive strategies such as avoidance and suppression is associated with impairing psychopathology such as depressive symptoms in adolescents (6).

Adolescence appears to be a critical time period for difficulties in ER due to endocrinological, cognitive and socio-emotional changes and challenges, e.g., emotional separation from the parents, first sexual encounters and higher social stressors due to a greater importance of the peer group (6). This leads to a higher frequency and intensity of emotions in adolescence compared to childhood, and an increased demand to regulate emotions (25). Given that cognitive skills, especially executive functions, are not fully developed in adolescents, cognitive strategies (such as reappraisal and problem solving) may be less accessible for children and adolescents (6). In addition, as mentioned above sleep problems impair executive functions (17) which in turn might reduce the ability to regulate emotions. As a consequence, an increased use of maladaptive strategies may enhance the development of depressive symptoms, whereas the use of adaptive strategies may be protective against the development of psychopathology. The increased demand to regulate emotions in adolescence on the one hand, and difficulties in ER on the other hand, may contribute to the emergence of depressive disorders during adolescence (26, 27). ER deficits may pose a risk factor for adolescent psychopathology. For example, a meta-analysis showed that rumination as one maladaptive strategy was a significant predictor of depressive symptoms (28).

Furthermore, a meta-analytic review of studies encompassing school samples revealed an association between the habitual use of adaptive or maladaptive strategies and depressive symptoms (i.e., fewer symptoms for adaptive strategies; *r* = −0.35, more symptoms for maladaptive strategies; *r* =0.42) (6). Also in adult samples, the relation between maladaptive ER and depressive symptoms emerged in a sample of diagnosed depressive patients (22). In particular, the use of maladaptive strategies was more important for psychopathology than the non-use of adaptive strategies. However, evidence among youth with clinical relevant levels of depression is scarce.

### Relationship Between Sleep and ER

**Figure 1:**
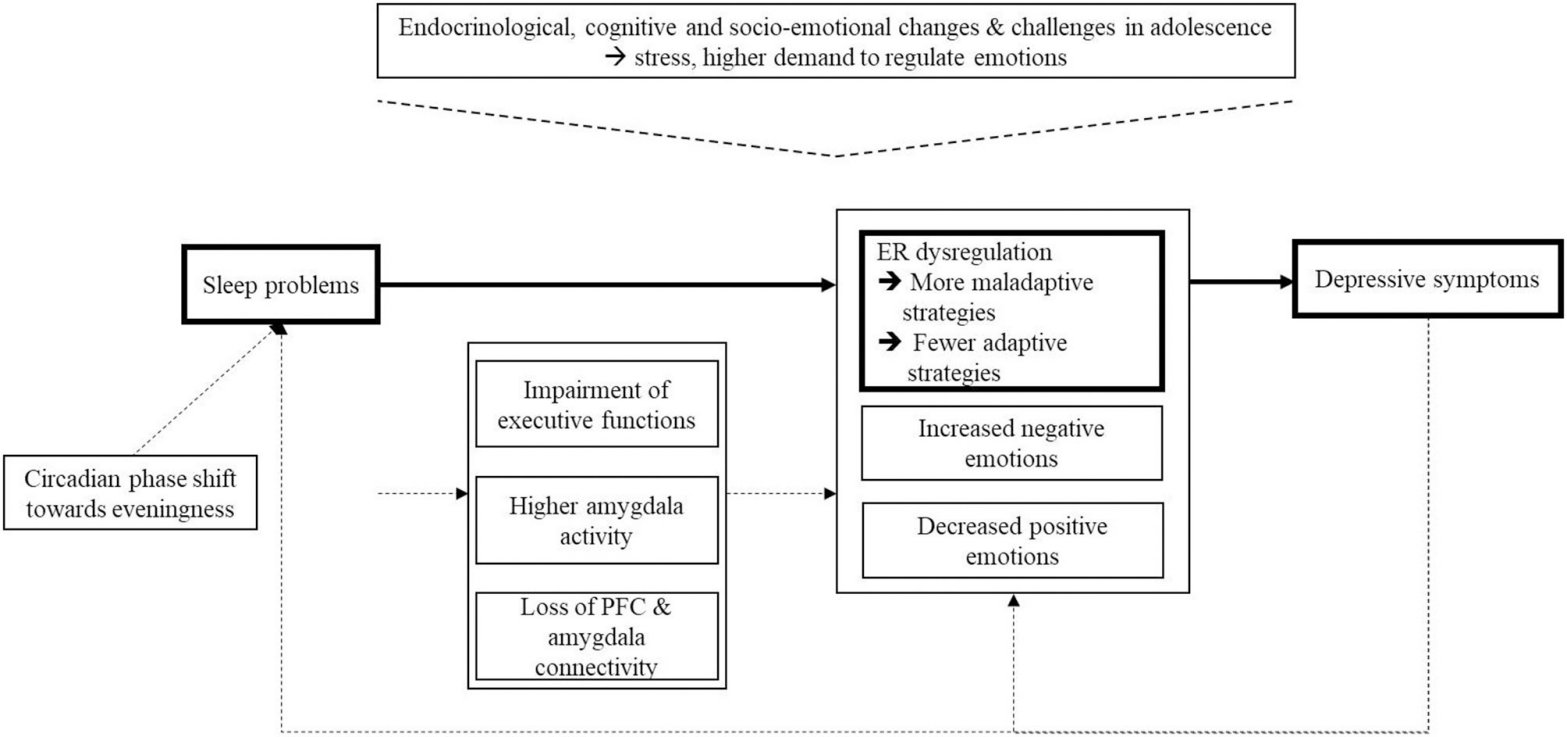
Schematic overview of the assumed associations and possible underlying mechanisms which are discussed in the literature. Dash line, evidence of prior literature; Solid line, associations under examination in the present study.

Studies exploring the relationship between sleep and ER found that experimentally induced poor sleep led to deficits in ER in adolescents and adults (29, 30). After sleep deprivation, participants reported a greater intensity of negative emotions, such as being more anxious, angry and irritable, and more difficulties in ER. Neuroimaging studies indicate that brain regions involved in ER are influenced by poor sleep (31, 32). Yoo et al. (32) investigated the effect of sleep deprivation on an emotional stimulus viewing task by using functional magnetic resonance image (fMRI) in young adults. The authors examined the activation of and the connectivity between the amygdala and the medial-prefrontal cortex (MPFC), both of which play an important role in the processing of emotional information (33). The results revealed higher amygdala activation and a loss of connectivity between the amygdala and the MPFC in a sleep-deprivation group compared to a sleep-control group (32), suggesting a higher amygdala response to negative emotional stimuli and a failure of top-down control after sleep deprivation. Figure 1 illustrates the assumed associations and summarized possible underlying mechanisms which were discussed in the literature (as described above).

The present study aims to replicate the reported mediation of ER dysfunction on the association of sleep problems and depressive symptoms in a clinical sample of adolescents with diagnosed depression (4). Dysfunctional ER is defined as increased use of maladaptive strategies to regulate emotions and a decreased use of adaptive strategies. Thus, we expect high correlations between poor sleep, depressive symptoms and an increased use of maladaptive strategies and a decreased use of adaptive strategies. To investigate differences between age and gender, these variables are examined as continuous covariates in the analyses. In addition, we compare two age groups—early adolescence (age 13–15) and late adolescence (age 16–18)—regarding the extent of sleep problems, depressive symptoms and ER strategies. Due to an increased circadian phase shift and higher emotional challenges in adolescence, we assume more severe symptoms in older adolescents. According to prior literature, we hypothesize that girls will show greater sleep problems, depressive symptoms and dysfunctional ER.

## Materials and Methods

### Sample

**Figure 2:**
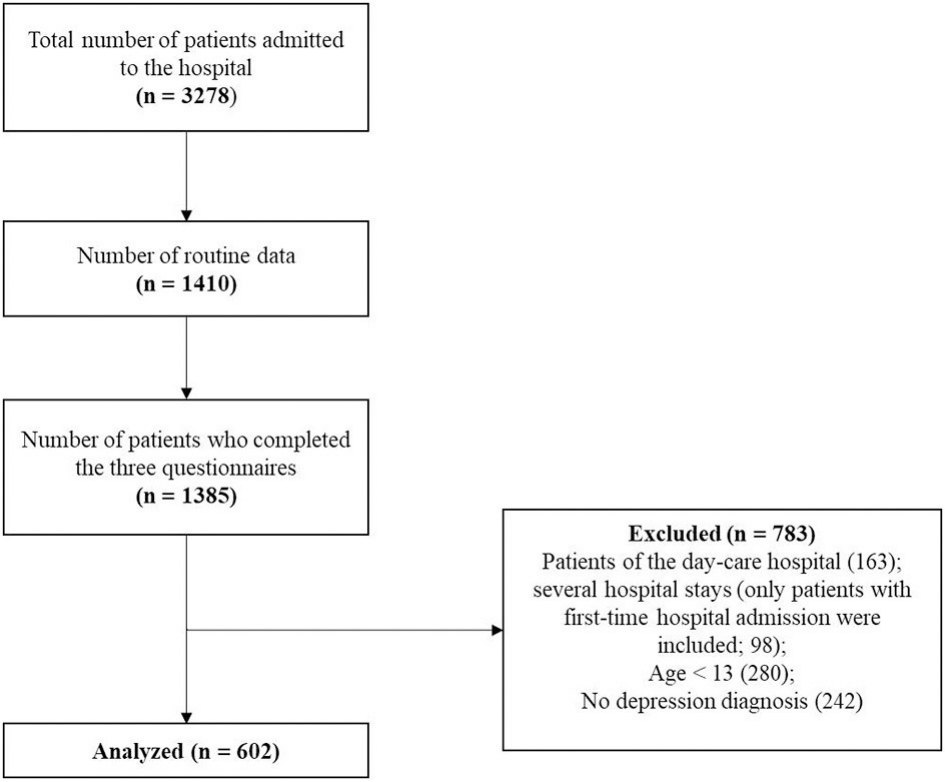
Patient flow.

Routine data, including questionnaires assessing sleep problems, depressive symptoms and ER were collected from all patients (aged 13–18 years) who were admitted to the LWL-University Hospital Hamm, Germany from 01/2016 to 06/2018. The hospital provides child and adolescent psychiatric care in an area with a population of 1.5 million inhabitants covering both urban and rural areas. It is the sole provider of inpatient child and adolescent psychiatric care for the study area. The final sample comprised 602 inpatients, of whom 421 (69.9%) were female. The mean age of the sample was 15.16 years (SD = 1.32). All included inpatients were diagnosed by clinicians with a depressive disorder or a depressive conduct disorder (inclusion criteria), which was the main diagnosis for 542 (90%) of the participants (F32.0; F32.1; F32.2; F32.3; F33.0; F33.1; F33.2; F33.3; F92.0; ICD-10). 78 of the 602 inpatients were diagnosed by clinicians with an anxiety disorder. 238 (39.5%) of the inpatients were diagnosed with one comorbid diagnosis, 90 (15%) with two comorbid diagnoses and 48 (8%) with more than two diagnoses. 226 (37.5%) were diagnosed with one diagnosis. The scientific use of the routine data was approved by the ethical review board of the Ruhr-University Bochum (registration number: 4359-12). All participants completed questionnaires on a laptop and were supported by a psychologist to provide the opportunity to ask questions if necessary. Details of the flow of participants are provided in Figure 2.

### Measures

#### ER

ER was assessed using the German-language Questionnaire on ER Strategies in Children and Adolescents (Fragebogen zur Erhebung der Emotionsregulation bei Kindern und Jugendlichen; FEEL-KJ) (34). The FEEL-KJ is a self-report questionnaire assessing different ER strategies in response to the emotions anger, sadness and anxiety. It consists of 90 items and was developed for children and adolescents aged from 10 to 20 years. For each of the three emotions, both adaptive and maladaptive strategies are assessed with two items each. Adaptive strategies are Problem Solving (e.g., “I try to change what makes me sad.”), Distraction (e.g., “I do something fun.”), Lightening the mood (e.g., “I think about cheerful things.”), Acceptance (e.g., “I accept what makes me sad.”), Forgetting (e.g., “I try to forget what makes me sad.”), Reappraisal (e.g., “I tell myself it is nothing important.”) and Cognitive Problem Solving (e.g., “I think about how I can solve the problem.”). Maladaptive strategies are Giving up (e.g., “I do not want to do anything.”), Aggressive Action (e.g., “I start a quarrel with someone.”), Withdrawal (e.g., “I do not want to see anyone.”), Self-Devaluation (e.g., “I blame myself.”) and Perseveration (e.g., “I keep thinking about why I am sad.”). These strategies are subsumed into two subscales, “adaptive strategies” and “maladaptive strategies.” Each item is rated on a 5-point scale from almost never (1) to almost always (5). Sum scores range from 42 to 210 for adaptive strategies and from 30 to 150 for maladaptive strategies. Norm values are indicated with *T*-values. For adaptive strategies *T*-values <40 are conspicuous (less use of adaptive strategies) and for maladaptive strategies *T*-values > 60 are conspicuous (often use of maladaptive strategies). The FEEL-KJ has shown good internal consistencies, with Cronbach's α = 0.93 and a test-retest reliability of *r*_tt_ =0.81 for adaptive strategies and Cronbach's α = 0.82 and a test-retest reliability of *r*_tt_ =0.73 for maladaptive strategies (34). Construct validity between CERQ (Cognitive Emotion Regulation Questionnaire for Children) (35) and the FEEL-KJ was investigated in a large sample of Dutch-speaking Belgian children and adolescents (*N* = 1102) (36). The correlations revealed a strong positive relation between the adaptive emotion regulation scales of these questionnaires (*r* = 0.67, *p* < 0.001) and a positive relation between the maladaptive scale of the FEEL-KJ and the Non-Adaptive-Emotion-Regulation scale of the CERQ (*r* = 0.36, *p* < 0.001).

#### Sleep Habits

The German-language Sleep Inventory for Children and Adolescents (Schlafinventar für Kinder und Jugendliche; SI-KJ) (37) is a 28-item questionnaire to assess the sleep habits of children and adolescents aged 5–18 years over the past 3 months. To assess sleep problems, the two subscales “difficulties in initiating and maintaining sleep” (e.g., “In the evening I have a hard time falling asleep.”) and “daily well-being” (e.g., “During the day, I am tired at school or when I am playing.”) were used in the present study. The items are measured on a 3-point Likert-scale ranging from 0 (not applicable) to 2 (exactly or frequently applicable). Sum scores range from 0 to 12. The sum score divided by the number of items ≥ 1 is defined as a cut-off for sleep problems. The internal consistency of these items in the current sample was Cronbach's α = 0.76. As norms are only available for children aged 8–11 years, sum scores were used for the statistical analyses.

#### Depressive Symptoms

Depressive symptoms were assessed with the German-language Depression Inventory for Children and Adolescents (Depressions-Inventar für Kinder und Jugendliche; DIKJ) (38). The DIKJ is a 26-item self-report questionnaire assessing the severity of depressive symptoms. It covers all relevant DSM-5 criteria for a depressive episode in a manner suitable for children and adolescents from the age of 8 years (39). Sum scores range from 0 to 50. The presence of clinically relevant depressive symptoms is indicated by a *T*-value > 60. The DIKJ has shown good psychometric properties, with Cronbach's α = 0.92 in a clinical sample (38). The internal consistency in the current sample was Cronbach's α = 0.86. To minimize item content overlap between the SI-KJ and the DIKJ, the sleep item was removed from the depression scale; therefore, sum scores were also used for the statistical analyses.

### Statistical Methods

All analyses were performed using SPSS 26.0 for Windows. The two-sided level of significance was set at α =0.05. To investigate direct and indirect effects between sleep problems and depressive symptoms, and the use of adaptive and maladaptive ER strategies as possible mediators, a regression analysis using PROCESS (40) was conducted. The regression model with depressive symptoms as dependent variable included the z-transformed sum score of the SI-KJ as independent variable and z-standardized FEEL-KJ total scores of adaptive and maladaptive ER strategies as mediators. Based on sample size calculations for mediation analyses using bootstrapping of Fritz and Mac Kinnon (41), for assumed weak pathways of the mediation model a sample size of 558 participants is needed. Age and gender were entered as covariates. Bootstrapping was performed with 10,000 samples. Independent *t*-tests were performed for the variables age (age <16 and age ≥ 16) and gender (girls and boys). Correlation analyses were conducted between the three variables ER (adaptive and maladaptive strategies; measured with the FEEL-KJ, total score) and sleep problems (two subscales measured with the SI-KJ) as well as depressive symptoms (measured with the DIKJ; total score without the sleep item). Furthermore, these variables were correlated to age (Pearson correlation) and gender (Spearman's rank order correlation).

## Results

### Sample Description

Table 1 presents means and standard deviations (SD) for sleep problems, depressive symptoms, and maladaptive and adaptive strategies for the total sample, for boys and girls and the two age groups separately. Independent *t*-tests revealed significant differences between girls and boys, with girls showing more sleep problems and depressive symptoms as well as a greater use of maladaptive strategies and a reduced use of adaptive strategies compared to boys. There was no significant effect of age. Clinically relevant depressive symptoms were reported by 74.8% of the sample. 78% of the sample showed conspicuous values for adaptive strategies and 61.1% for maladaptive strategies. 74.5% of the sample reported clinically relevant sleep problems.

**Table 1:** Sample characteristics (sum scores, *T-*values in brackets) regarding sleep, emotion regulation and depressive symptoms.

	**Group**			**Test statistics**		**Group**		**Test statistics**	
	**Total sample *N* = 602**	**Boys *N* = 181**	**Girls *N* = 421**			**Age <16 *N* = 346**	**Age ≥ 16 *N* = 256**		
				***t***	***p***			***t***	***p***
DIKJ									
M	23.85	20.00	25.51	*t*_(600)_ = −7.694	<0.001	24.17	23.42	*t*_(589)_ = 1.113	0.266
(*T*-value)	(68.01)	(62.49)	(70.38)			(67.95)	(68.08)		
SD	8.44	7.90	8.12			8.99	7.63		
(*T*-value)	(11.12)	(11.06)	(10.28)			(11.49)	(10.62)		
SI-KJ									
M	7.40	5.86	8.06	*t*_(594)_ = −8.484	<0.001	7.34	7.49	*t*_(594)_ = −0.590	0.555
SD	3.07	3.03	2.85			3.16	2.94		
FEEL-adaptive									
M	106.53	113.32	103.61	*t*_(600)_ = 3.936	<0.001	106.4	106.7	*t*_(600)_ = −0.128	0.898
(*T*-value)	(36.65)	(38.83)	(35.72)			(36.62)	(36.70)		
SD	28.09	29.02	27.21			28.63	27.40		
(*T*-value)	(12.41)	(13.34)	(11.88)			(12.74)	(11.93)		
FEEL-maladaptive									
M	96.68	87.84	100.48	*t*_(600)_ = −7.725	<0.001	95.4	98.41	*t*_(589)_ = −1.947	0.052
(*T*-value)	(62.26)	(55.71)	(65.08)			(61.24)	(63.64)		
SD	19.29	19.23	10.40			20.49	17.41		
(*T*-value)	(16.47)	(16.89)	(15.48)			(17.22)	(15.32)		

*DIKJ, Depression Inventory for Children and Adolescents; SI-KJ, Sleep Inventory for Children and Adolescents; FEEL-adaptive, German Questionnaire on Emotion Regulation Strategies in Children and Adolescents – subscale “adaptive strategies”; FEEL-maladaptive, German Questionnaire on Emotion Regulation Strategies in Children and Adolescents – subscale “maladaptive strategies”*.

### Correlations Between Adaptive and Maladaptive Strategies, Sleep Problems and Depressive Symptoms

Depressive symptoms and sleep problems were significantly correlated (*r* = 0.592, *p* < 0.001). The use of adaptive ER strategies was significantly associated with depressive symptoms (*r* = −0.489, *p* < 0.001) and with sleep problems (*r* = −0.373, *p* < 0.001). The use of maladaptive ER strategies was also significantly associated with depressive symptoms (*r* = 0.655, *p* < 0.001) and with sleep problems (*r* = 0.623, *p* < 0.001).

### Mediation by Adaptive and Maladaptive ER Strategies

**Figure 3:**
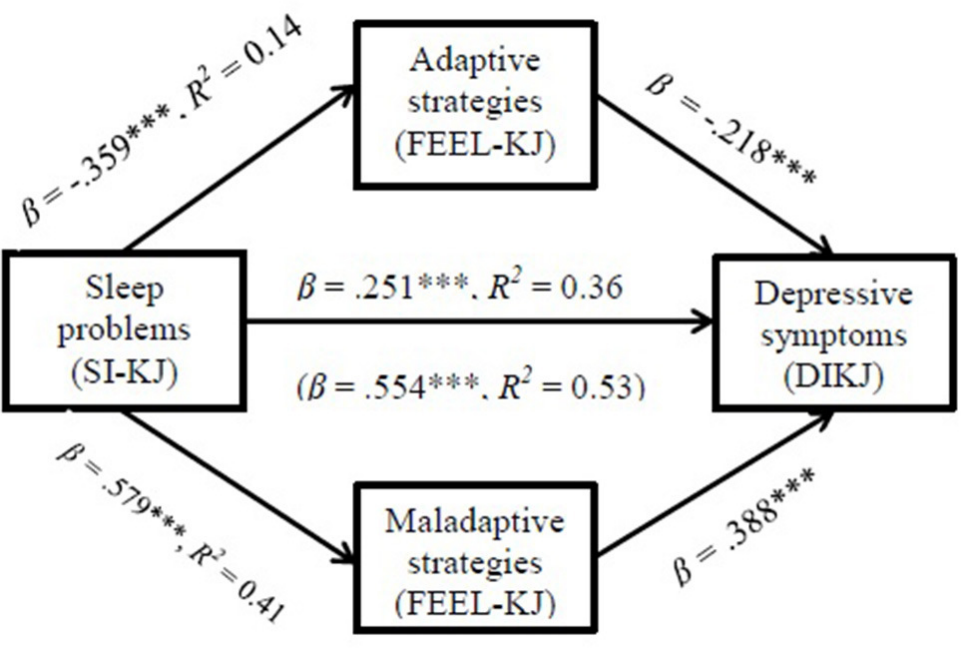
Indirect effects of adaptive and maladaptive strategies on the relationship between sleep problems and depressive symptoms. SI-KJ Sleep Inventory for Children and Adolescents; FEEL-KJ German Questionnaire on Emotion Regulation Strategies in Children and Adolescents; DIKJ Depression Inventory for Children and Adolescents; date in brackets refer to the total effect of the model; ***p < 0.001.

The mediation analysis revealed a significant partial mediation model, with a positive standardized indirect effect on the relationship between sleep problems and depressive symptoms via the use of maladaptive strategies (β = 0.226, *CI* [0.177, 0.280]) and a negative standardized indirect effect via the use of adaptive strategies (β = 0.079, *CI* [0.051, 0.109]). The direct effect of sleep problems on depressive symptoms was weaker but still significant (β = 0.251, *t* = 6.765, *p* < 0.001, *CI* [0.178, 0.323], *R*^2^ = 0.364) compared to the total effect (β =0.554, *t* = 16.012, *p* < 0.001, *CI* [0.486, 622], *R*^2^ =0.527). Gender (*coefficient* = 0.256, *p* < 0.001, *CI* [0.112, 0.401]) and age (*coefficient* = 0.067, *p* = 0.0057, *CI* [0.020, 0.114]) were significant covariates for maladaptive strategies. For depressive symptoms, age was a significant covariate in the total effect model (*coefficient* = −0.052, *p* =0.017, *CI* [−0.094, −0.009]) and gender in the direct effect model (*coefficient* = 0.239, *p* = 0.002, *CI* [0.089, 0.388]). The mediation model is illustrated in Figure 3.

## Discussion

In line with our assumptions and prior literature in adults (4), there was a significant partial mediation effect, insofar as sleep problems influenced depressive symptoms via dysfunctional ER. In contrast to adults, not just the increased use of maladaptive strategies but also the decreased use of adaptive strategies partly mediated the association between sleep problems and depressive symptoms. Additionally, a direct effect of sleep problems on depressive symptoms emerged. In this clinical sample, sleep problems were positively correlated with depressive symptoms as well as maladaptive ER strategies and negatively correlated with adaptive ER strategies. Furthermore, depressive symptoms were associated with an increased use of maladaptive strategies and with a decreased use of adaptive strategies. In addition, gender was a significant covariate in the present study, whereas age was not.

### The Mediating Effects of Maladaptive and Adaptive Strategies on the Association Between Sleep and Depression

The present findings confirm previous suggestions that ER acts as a possible mechanism underlying the association between sleep and depression (4, 19). Sleep problems seem to influence ER negatively insofar as more maladaptive strategies and fewer adaptive strategies are used to regulate emotions, which in turn might facilitate the development of depressive symptoms. Interestingly, in the present study, the decreased use of adaptive ER strategies was also associated with psychopathology, and mediated the association between sleep problems and depressive symptoms. This is in line with the findings of Schäfer et al. (6), who reported negative associations between the habitual use of adaptive strategies and depressive symptoms in a meta-analysis of non-clinical school samples. In contrast, a meta-analytic-study in adults by Aldao and Nolen-Hoeksema (22) only revealed associations between maladaptive ER strategies and depressive symptoms. As a consequence, the authors concluded that the use of adaptive strategies seems less important for psychopathology than the use of maladaptive strategies. These conflicting findings may be explained by the different age groups of the respective samples (children and adolescents vs. adults). In sum, in contrast to adults, both adaptive and maladaptive strategies might be important for psychopathology in adolescents.

In accordance with our assumptions, poor sleep was associated with more depressive symptoms, and both poor sleep and depressive symptoms were correlated with an increased use of maladaptive strategies and a decreased use of adaptive strategies. Different underlying mechanisms for these associations have been discussed before. E.g., neuroimaging studies indicated that brain regions involved in ER, predominantly in the limbic system, were influenced by sleep deprivation and worse sleep (42). Sleep deprivation and worse sleep seem to interrupt the connection between the MPFC and amygdala, leading to difficulties in inhibiting negative emotions (32). A negative memory bias due to sleep problems has also been discussed as an underlying mechanism (19). Evidence suggests that insufficient sleep leads to difficulties in encoding positive and neutral emotional memories, whereas negative experiences can be remembered (19). Recently, evidence emerged that supports these assumptions: whereas differences between subjective reports of sleep disturbances between low and high familial risk for depressive disorders emerged, objective measurements, namely actigraphy and sleep diary-based measures of sleep duration and midpoint of sleep, revealed no significant differences between high and low risk adolescents. These differences in subjective and objective measures of sleep disturbances may reflect a potential cognitive bias (to negative information) in parents with depression and in adolescents with high risk for depression (16).

### Gender and Age as Covariates

As many studies showed that girls are two to three times more afflicted by depressive symptoms at the age of 13 or 14 than boys [e.g., (43)], it is not surprising that gender was a significant covariate in the present study. Furthermore, the results revealed that girls are more affected by depressive symptoms, sleep problems and emotion dysregulation. These findings are also in line with previous research [e.g., (16, 44)]. For example, Hankin (44) investigated cognitive vulnerabilities and stressful life events as mediators for sex differences in depressive symptoms during adolescence in a prospective, multiwave study. Therein higher levels of depressive symptoms in girls were mediated by a negative cognitive style x stressors interaction as well as rumination x stressors interaction. Girls reported higher levels of depressive symptoms, negative cognitive style, rumination and greater exposure to stressors than did boys. In sum, girls seem to be more afflicted than boys due to higher levels of stress in adolescence and a greater use of maladaptive strategies, which in turn leads to higher levels of depressive symptoms. The associations between sleep problems, the use of ER strategies and depressive symptoms seem to be more important for girls than for boys.

Age did not moderate these associations in this clinical adolescent sample, although older adolescents were more afflicted due to their greater use of maladaptive strategies. As the use of maladaptive strategies seems to be more important for psychopathology than the non-use of adaptive strategies in adulthood (22), it might be assumed that the positive association between age and maladaptive strategies correlates with the duration and chronicity of depression insofar as longer duration of depression (in adults and older adolescents) leads to a higher use of maladaptive strategies. However, as we have no information about the duration and chronicity of depression, we were unable to verify this hypothesis in the current sample.

### Limitations

The present study overcomes some of the limitations of previous studies by investigating the associations between sleep, depression and ER in a large clinical sample of adolescents with clinical levels of depression. However, limitations of this study are the cross-sectional design and the use of self-report assessment tools to reflect habitual use of ER strategies and sleep habits over time and across different contexts (22), which might have impacted the results and thus reduced the generalizability of the findings. Also the use of a clinical sample limits the generalizability of the results to population samples. Thus, the associations should be investigated in population-based samples. Further studies should also provide data on treatment course. As two-thirds of the inpatients were diagnosed with a comorbid psychiatric disorder, another limitation is that we did not controlled for the confounding bias of comorbid disorders, especially the influence of anxiety might be consider in further studies. Differences in the relevance of the non-use of adaptive strategies for psychopathology between adults and youth might be caused by longer depressive illness duration, thus a further limitation is that we were unable to control for the age of onset and duration of depression in the present analyses. Another limitation is that the study sample also included the heterogenous category F92.0 and not just depressive diagnoses. But the naturalistic design of the study and the clinical experience of the authors that especially younger adolescents which were diagnosed with F92.0 show high depressive symptoms led to the decision to include this diagnosis group. Due to the cross-sectional study design, the results should be interpreted with caution and should be seen as preliminary. Longitudinal and experimental studies are needed to replicate the results and to provide a more thorough understanding of the influence of sleep problems and ER deficits on depressive symptoms. In addition, further studies are needed to investigate the impact of further variables such as experiences of stress and a cognitive bias to negative information processing and to strengthen the findings of possible underlying mechanisms and their associations (see Figure 1).

### Implications and Contribution

Taken together, the present results indicate deficits in ER as a mechanism partly explaining the established relation between sleep problems and depressive symptoms. If longitudinal studies in adolescents with clinical levels of depression substantiate these findings the application of additional treatment modules focusing on sleep and ER skills in prevention and treatment programs for children and adolescents would be important steps. To reduce the risk of developing depressive symptoms, children and adolescents should acquire strategies to manage their emotions. There is already preliminary evidence that the application of ER prevention programs has positive effects on depressive symptoms. For example, adolescents with non-clinical depressive symptoms or clinically anxious youth were trained to use strategies of cognitive reappraisal, which led to a reduction in the frequency of negative emotions and improved self-reported ER (28, 45).

Due to the influence of sleep problems on depressive symptoms, the improvement of sleep could represent another treatment opportunity in the prevention and treatment of depression. In adults, evidence suggests that cognitive behavioral therapy for insomnia (CBT-I) reduces insomnia and depressive disorders to a greater extent than relaxation training (46). In addition, first evidence suggests that morning bright light therapy may be efficient as an add-on treatment option to reduce sleep problems in adolescent depression (47, 48).

## Data Availability Statement

The raw data supporting the conclusions of this article will be made available by the authors, without undue reservation.

## Ethics Statement

The studies involving human participants were reviewed and approved by Ethical review board of the Ruhr-University Bochum (registration number: 4359-12). Written informed consent for participation was not provided by the participants' legal guardians/next of kin because: Ethical approval for the routine data has been giving without written consent from all patients and their legal guardian. Participants consented to the study by completing the questionnaires. This procedure is regulated by law regarding using routine data in hospitals (Art. 9, Paragraph 2, Letter j DSGVO (data protection declaration of Germany) in relation to §5 Paragraph 5 DSG NRW(data protection declaration of Nordrhein-Westfalen) and §17 DSG NRW in relation to §6 Paragraph 2 GDSG (data protection law for health care) NRW. This procedure of the scientific data use of the routine data was approved by the ethical review board of the Ruhr-University Bochum (registration number: 4359-12).

## Author Contributions

IK-L, MH, and TL designed the study. IK-L managed the literature searches and analyses, undertook the statistical analyses, and wrote the first draft of the manuscript. All authors contributed to and have approved the final manuscript.

## Conflict of Interest

MH served in an advisory role and was paid for public speaking by Shire and Medice. He receives research support from the German Research Foundation and the German Ministry of Education and Research. He receives royalties as Editor-in-Chief of the German Journal for Child and Adolescent Psychiatry and for textbooks from Hogrefe. TL received conference attendance support from Lilly as well as royalties for textbooks from Hogrefe and Springer. The remaining author declares that the research was conducted in the absence of any commercial or financial relationships that could be construed as a potential conflict of interest.
